# Can consumers’ gamified, personalized, and engaging experiences with VR fashion apps increase in-app purchase intention by fulfilling needs?

**DOI:** 10.1186/s40691-021-00270-9

**Published:** 2021-10-05

**Authors:** Oiyan Lau, Chung-Wha (Chloe) Ki

**Affiliations:** 1 Master Student, Institute of Textiles and Clothing, The Hong Kong Polytechnic University, Hunghom, Kowloon, Hong Kong; 2 Assistant Professor, Institute of Textiles and Clothing, The Hong Kong Polytechnic University, Hunghom, Kowloon, Hong Kong

**Keywords:** Mobile commerce, Virtual reality (VR) mobile apps, 3D avatar-based VR, Game-based VR, Digital fashion, Digital clothing, In-app purchase

## Abstract

While the development of virtual reality (VR) apps is trending among fashion retailers to cope with the COVID-19 pandemic and promote consumers’ online shopping, less is understood about whether and the way these new VR apps increase consumers’ in-app purchase. This study was designed to address this issue by applying self-determination theory within the context of Taobao Life, a 3D avatar-based and game-featured virtual world on the Taobao app. Specifically, we investigated (1) whether the extent to which a VR fashion app provides consumers with a sense of gamified experience (H1: challenge, and H2: achievement), personalized experience (H3: avatar customization, and H4: avatar identification), and engaging experience (H5: social presence, and H6: social support) fulfills their competence, autonomy, and relatedness needs; (2) whether these intrinsic needs fulfill determine positive consumer behavioral intentions (H7: intention to continue to use VR apps, and H8: intention to make in-app purchase), and (3) whether the intention to continue to use VR apps leads to a positive in-app purchase intention (H9). We tested the above empirically by conducting an online survey via Dynata, and the dataset of 251 responses was analyzed using structural equation modeling. The findings of our research provide theoretical and practical implications that can be applied in the fashion retail business.

## Introduction

The COVID-19 pandemic is reshaping the retail landscape and has accelerated its pace of digital transformation. Hit hard by the pandemic and social unrest, fashion retailers have been forced to adapt to the new retail normal to rescue their businesses (Ng, 2020). One way to do so was by shifting their focus from conventional brick-and-mortar retail operations to mobile commerce (m-commerce) to increase online sales (Kohan, 2020). M-commerce refers to any commercial transaction completed with a mobile device—whether a smartphone or tablet—which results in the transfer of value in exchange for services or goods (Magrath & McCormick, 2013). Examples of m-commerce include in-app purchasing (i.e., buying goods and services within an application on a mobile device), mobile banking, or using a digital wallet (e.g., Apple Pay or PayPal).

Mobile apps had already made significant market inroads in the fashion retail business before the COVID-19 pandemic (Torok, 2020). However, the recent coronavirus crisis has supercharged them even further as fashion retailers look for ways to enhance consumer e-commerce connections. In fact, 40% of consumers who did not shop online previously have begun to use e-commerce channels during the pandemic and 26% expect to shop less at physical stores following COVID-19 (Barbiroglio, 2020). During the first half of 2020, the e-commerce market in the U.S. increased by 30% compared to the same period in 2019 (Rabimov, 2020). In particular, mobile retail spending amounted to over US $47.8 billion during the second quarter of 2020 (Statista, 2020). Experts forecast that by 2021, 53.9% of the annual retail e-commerce sales in the U.S. will derive from m-commerce (Business Wire, 2018).

As m-commerce’s popularity grows, technological advancements, such as virtual reality (VR), augmented reality (AR), and 3D visualization, are being applied increasingly in fashion mobile apps to offer extra features that appeal to customers (Kohan, 2020). In today’s post-pandemic reality where consumers demand physically-disconnected, yet emotionally-connected interactions with fashion brands, it is particularly important for retailers to engage customers in the new VR normal via their apps. Thus, an increasing number of fashion retailers has developed, or accelerated their development of, VR apps to engage with consumers during the lockdown (Morgan, 2020). For example, the Chinese retail giant, Alibaba, recently launched a 3D avatar-based virtual world in their Taobao app that features a game—Taobao Life. In Taobao Life, users can customize their avatars and navigate a game-like virtual world with them. Further, the U.S. retail giant, Amazon, is developing a VR app referred to as a ‘virtual fitting room’ that will allow consumers to try on outfits with a customized virtual model of themselves (Petro, 2021).

While VR apps have emerged as one of the newest trends in the fashion retail business, less is understood about consumers’ perceptions of, and experiences with, these novel apps (Parker & Wang, 2016). This led us to an important question: do fashion retailers’ VR apps increase online sales, and if so, how? To generate a more specific and meaningful set of research questions, we review the literature on fashion mobile apps in greater detail in the section that follows, which helped us gain a clearer understanding of what has been explored versus underexplored in the literature.

## Literature review

### Fashion mobile apps

Retail operations are becoming increasingly mobile, and the fashion industry is leading the way, with the majority of online fashion sales now made on mobile devices, particularly through mobile apps (Charlton, 2019). Fashion mobile apps offer a convenient and user-friendly way for consumers to browse and purchase fashion items (Magrath & McCormick, 2013). Because of mobile apps’ exponential growth and wide adoption on the part of fashion retailers and marketers, academic researchers have devoted much attention to this topic. In doing so, much of the former research has focused on identifying the distinct attitudes consumers display (e.g., consumers’ adoption intention or their feelings of satisfaction) in response to mobile app use, and the specific factors that affect those attitudes. For example, Hur et al.’s (2017) study investigated the factors that influence consumers’ adoption of fashion mobile apps. In doing so, they identified technological innovativeness (i.e., individual propensity to try new technology) and fashion innovativeness (i.e., individual propensity to purchase new fashion items rather than staying with previous choices and/or consumption patterns) as the critical antecedents that influence consumers’ fashion app adoption. On the other hand, Trivedi and Trivedi (2018) explored the factors that affect consumers’ satisfaction with fashion mobile apps, and documented that three factors, information quality (the extent to which consumers find that fashion mobile apps provide quality information), system quality (the extent to which consumers find fashion mobile apps systematically easy to use), and service quality (the extent to which consumers find that fashion mobile apps provide good after-sales services), affect consumer satisfaction with fashion mobile apps significantly.

Despite these previous studies’ theoretical contributions, their practical implications are limited, in that many of them have considered ‘attitude’ the most important predictor of consumer behaviour. Indeed, understanding consumers’ attitude, which encompasses consumers’ beliefs, feelings, or behavioural intentions toward an attitude object, is important in fashion marketing research. However, in today’s dynamic fashion business environment where consumers’ tastes and preferences are changing constantly, it seems more important to understand whether and the way fashion mobile apps satisfy consumers’ more enduring, intrinsic needs (e.g., for competence, autonomy, and relatedness), which are essential in motivating their behaviour. Further, as mobile apps have advanced technologically over time and in the unique pandemic context, it is important to understand whether and the way the new ‘VR’ fashion apps that are endowed with gamification-, customization-, and engagement-features fulfill consumers’ intrinsic needs for competence, autonomy, and relatedness. This led us to formulate the following research questions:

- RQ1: Do the gamification-, customization-, and engagement-elements of VR fashion apps satisfy consumers’ intrinsic needs for competence, autonomy, and relatedness, respectively?
- RQ2: If so, does this fulfill of intrinsic needs (i.e., competence, autonomy and relatedness) shape consumers’ positive behavioural intention (i.e., the intentions to continuously use the VR app and make in-app purchases)?

To address the questions above, we developed our conceptual model based upon self-determination theory (SDT).

### Self-determination theory (SDT)

SDT is a theory of human motivation Deci and Ryan (1985) developed. In this context, motivation refers to what moves people to act. *Self-determination* represents an individual’s ability to make choices and determine a course of action according to his/her own will, without external compulsion (Wehmeyer & Little, 2013). This ability plays an important role in motivating humans’ behavior and enhancing their psychological wellbeing (Ki & Kim, 2016). Self-determination allows people to feel that they have control over their choices and lives, which ultimately increases their feelings of psychological wellbeing (e.g., feeling capable, self-governed, well-supported, and satisfied with their state) (Ryan, 2009). It also has an influence on motivation. For example, people will feel more motivated to take action when they feel that what they choose to do on their own will have an effect on their outcome (Gagné & Deci, 2005). In this way, SDT highlights the essential role intrinsic motivation plays in affecting human behavior (Ki & Kim, 2016).

Stated differently, SDT examines people’s inherent tendencies that motivate them to engage in behaviors (Deci & Ryan, 2008). According to SDT, three basic psychological needs are particularly important in motivating a person to initiate behavior; an individual can become self-determined when his/her needs for *competence, autonomy*, and *relatedness* are fulfilled (Ryan & Deci, 2000a). Competence refers to the human need to feel that one’s behavior is enacted effectively [e.g., to feel that one has done a good job: Ryan and Deci (2000b)]. Autonomy represents the need to experience behavior as voluntary [e.g., to feel that one has control over what s/he does: Ryan et al., (2006)]. Relatedness refers to the need to interact, be connected to, and experience caring from others [e.g., to feel that one has meaningful relationships and interactions with others: Ryan and Deci (2000b)]. These three needs promote intrinsic motivation, which initiates behavior for its own sake and enhances one’s growth.

SDT has been applied in a wide range of research across distinct domains, from education to work and health (Gagné & Deci, 2005; Hagger & Chatzisarantis, 2009; Reeve, 2002). For example, it has provided a framework for academic researchers to identify ways to motivate students, employees, or patients better to enhance their academic, work, or exercise performance—particularly to foster their intrinsic motivations. While a number of studies has proposed *self-determined motivation* as an important predictor of human behaviour (Darner, 2009; Deci & Ryan, 2002; Wilson et al., 2008), academic researchers in the field of fashion marketing (i.e., fashion consumer behaviour) have been relatively slow to adopt SDT. Instead, they have been influenced largely by the theory of reasoned action (Ajzen & Fishbein, 1980) and have proposed ‘attitude’ (Choi & Lee, 2019; Kim & Karpova, 2010) and/or ‘subjective norm’ (Nam et al., 2017; Ramkumar & Woo, 2018) as important predictors of fashion consumer behaviour. However, more recently, SDT’s concept of autonomous motivation (i.e., basic and intrinsic human needs) has been shown to predict consumer behaviour better than its more established predictors (e.g., ‘attitude’, ‘subjective norms’, and/or ‘past behaviour’) (Gilal et al., 2018, 2019; Xi & Hamari, 2019). Thus, this study adopts this more recent stance and investigates (1) whether the extent to which a VR fashion app provides consumers with a sense of gamified experience (H1: challenge, and H2: achievement), personalized experience (H3: avatar customization, and H4: avatar identification), and engaging experience (H5: social presence, and H6: social support) fulfills their competence, autonomy, and relatedness needs; (2) whether these intrinsic needs fulfill determine positive consumer behavioral intentions (H7: intention to continue to use VR apps, and H8: in-app purchase intention), and (3) whether the intention to continue to use VR apps leads to a positive in-app purchase intention (H9).

### Hypotheses development

#### A VR fashion app’s gamification elements affect consumers’ need to fulfill competence

Gamification refers to a strategy of incorporating game-like elements (e.g., engaging in entertaining challenges and accomplishing distinct achievement levels) into a brand’s retail marketing programs (Singh, 2012). For example, in a VR fashion app such as Taobao Life, users can complete daily challenges and attempt to earn higher points with which they can buy virtual clothes or accessories (Hallanan, 2020). Fashion retailers are integrating these game dynamics into their mobile apps increasingly, as they believe they can satisfy consumers’ intrinsic need for competence (Xi & Hamari, 2019), which can be fulfilled particularly when they sense a degree of *challenge* (e.g., gradually improve through the course of the game) and *achievement* (e.g., achieve higher levels or points) while using VR apps (Sailer et al., 2017).

A sense of challenge refers to an individual’s feeling of engaging in a difficult, yet achievable, task (Fu et al., 2009). This sense of challenge has been found to satisfy consumers’ desire for competence effectively. Legault’s (2017) study showed that people prefer tasks that are more, rather than less, challenging. The more challenging the tasks, the more they require people’s attention, persistence, and determination to improve, all of which serve to fulfill their need to be competent. Facing and overcoming challenges indeed reinforce one’s beliefs in self-efforts and self-abilities, and thus fulfill the need for competence (Skhirtladze et al., 2019). This suggests that consumers’ competence need can be fulfilled by the challenge features designed in a VR app (e.g., passing different levels in tile-matching games on a VR fashion app such as Taobao Life). This leads us to formulate:

##### H1

The more consumers feel challenged while using a VR fashion app, the more they feel that their need for competence is fulfilled.

Providing consumers with a sense of achievement is arguably another important element that can fulfill their need for competence. A sense of achievement refers to a strong positive feeling (i.e., feeling proud) of having done something difficult and worthwhile (Merriam-Webster, n.d.). When a user experiences a sense of self-efficacy (i.e., the belief that s/he has the ability to accomplish a task) or a sense of mastery, it helps fulfill his/her need for competence (Skhirtladze et al., 2019). The need for competence can be fulfilled when people feel that they are capable of, or effective in, their actions (Sheldon et al., 2001). Excelling and gaining mastery over challenges can also allow people to gain an important sense of competence and develop a cohesive sense of self (Patrick et al., 2007). We expect that the same effect will hold true when consumers feel a sense of achievement while using a VR fashion app. This leads us to propose:

##### H2

The more consumers feel a sense of achievement while using a VR fashion app, the more they feel that their need for competence is fulfilled.

#### A VR fashion app’s customization elements affect consumers’ need to fulfill autonomy

Customization refers to a marketing strategy in which a retailer allows customers to individualize its products or services through personal engagement (Liao et al., 2019). Fashion retailers are integrating customization into their mobile apps increasingly to co-create value with their customers and integrate those added values into their offerings. For example, an American fashion retailer, The Gap, offers a mobile app, the ‘DressingRoom’, in which consumers can customize their avatars based upon their own body type and try on clothes virtually (Mileva, 2019). In this way, customers are given the autonomy to personalize fashion retailers’ mobile services.

Many previous studies have shown that avatar customization helps consumers fulfill their need for autonomy. Avatar customization is a feature that allows consumers to choose, design, or modify their avatars (Ratan & Sah, 2015). When consumers have the freedom to personalize something, they feel their intrinsic needs fulfilled (Ryan & Deci, 2000b). More specifically, the more people are given numerous options and the freedom to make their own choice, the more they feel their autonomy is enhanced (Kim et al., 2015). In contrast, the more they lack choices or control over things, the more they feel their autonomy is diminished (Kim et al., 2015). Hanus and Fox (2015) also indicated that people gratify their need for autonomy when they are given the freedom to customize something. This led us to formulate:

##### H3

The more consumers are able to customize their avatars on a VR fashion app, the more they feel their need for autonomy is fulfilled.

Avatar identification is another feature of a VR fashion app that allows consumers to enjoy a personalized experience and experience a feeling of autonomy. Avatar identification refers to the degree to which consumers perceive that an avatar is similar to them (Teng, 2019). For example, in the online gaming context, many previous studies have found that players tend to identify psychologically with their avatars and thereby project some aspects of their real-life identities onto those avatars while playing games (Li et al., 2013; Sioni et al., 2017). Particularly, when an avatar’s visual features, such as the face, skin color, hairstyle, clothes, and accessories, resemble those of the game player, s/he tends to identify those avatars as an extension of him/herself (Teng, 2019). This sense of identification with the avatar has been documented to enhance the game players’ need for autonomy significantly (Kao, 2019). Bailey et al.’s (2009) study also showed that the more one identifies an avatar as a representation of him/herself and feels ownership over it, the greater the propensity to feel his/her need for autonomy is satisfied. Thus, we propose:

##### H4

The more consumers identify their avatars on a VR fashion app as a representation of themselves, the more they feel that their need for autonomy is fulfilled.

#### A VR fashion app’s engagement features affect consumers’ need to fulfill relatedness

In fashion marketing, customer engagement refers to an interaction between a fashion retailer and its consumers (Pansari & Kumar, 2017), or as the means by which a fashion retailer creates a relationship with its customers to foster brand resonance (So et al., 2016). Fashion brands are incorporating this engagement strategy into their digital marketing programs increasingly, with the goal to offer consumers an interactive experience in the digital realm. This interactive experience can be shaped by providing consumers with a sense of social presence (Chattaraman et al., 2012) and social support (Shim et al., 2012).

In our context, social presence refers to the degree to which one perceives the presence of others while using a VR app (Mäntymäki & Salo, 2010). Social presence has been discussed as an important element that fosters interactivity in online media contexts, including websites, social media, and online gaming platforms (Karapanos et al., 2016). Many previous studies have documented the significant and positive relation between social presence and relatedness need fulfillment (Gao et al., 2018; Tseng et al., 2019). Presence signalling (e.g., social presence in a virtual world) was found to help people gratify their desire to feel connected to and develop meaningful relationships with others (Halfmann & Rieger, 2019). For example, in an online gaming context, when game players perceived a greater sense of social presence from their co-players, their relatedness need fulfillment was enhanced (Bormann & Greitemeyer, 2015). In a similar vein, in an online learning context, the more learners exchanged opinions with their peers online and experienced social interaction and social presence, the greater they felt their need for relatedness satisfied (Fang et al., 2019). We expect that this effect of social presence on relatedness need fulfillment will hold true in consumers’ VR app use. Thus, we postulate:

##### H5

The more consumers feel social presence while using a VR fashion app, the more they feel their need for relatedness is fulfilled.

Social support is another important element that can gratify consumers’ intrinsic need for relatedness. In our context, social support refers to the perception that a consumer is cared for and is part of a supportive social network while using a VR fashion app. Studies have shown that social support fosters people’s sense of belonging and thus, relatedness (Hagger et al., 2006; Hombrados-Mendieta et al., 2013). Bryan et al. (2016) indicated that relatedness is satisfied when people feel connected to, and supported by, others. Further, Reis et al.’s (2000) study showed that one of the strongest predictors of relatedness is feeling understood, which could be fostered by individuals’ perceptions of social support. In further support, Niemiec et al. (2014) showed that the more individuals perceive social support, the more they believe that they are having an authentic relationship with others, which satisfies their relatedness need. This led us to propose:

##### H6

The more consumers feel social support while using a VR fashion app, the more they feel their need for relatedness is fulfilled.

#### Consumers’ intrinsic needs fulfillment affects their behaviour positively

Consistent with SDT’s core premise, many previous studies have indicated that individuals’ needs fulfillment is a critical motivator of their behavior (Bhattacherjee, 2001; Vansteenkiste et al., 2005). Here, we discuss further the way people’s intrinsic needs fulfillment activates their behavioral intention (i.e., the intention to use the app continuously and to make in-app purchases). First, many former studies across diverse virtual contexts, including the e-learning and online gaming contexts, have found the effects of competence, autonomy, and relatedness needs fulfillment on continuous use intention (Fang et al., 2019; Liao et al., 2020). In an e-learning context, when students perceived that e-learning enhanced their desire to perform efficiently and effectively (i.e., to be ‘competent’), they expressed genuine interest in e-learning and showed the intention to learn continuously (Sørebø et al., 2009). Continuous use intention can also be promoted when individuals’ needs for autonomy and relatedness are fulfilled. In a virtual gaming context, the more individuals are satisfied because of their freedom to make self-directed decisions while playing games (e.g., the ‘autonomy’ to customize their game avatars), the more likely they are to play the game continuously (Yoo et al., 2013). Further, the more people develop meaningful emotional bonds with other players and fulfill their desire for ‘relatedness’, the more they play the game again (Liao et al., 2020). This led us to propose:

##### H7

The more consumers feel their needs for (a) competence, (b) autonomy and (c) relatedness are fulfilled while using a VR fashion app, the greater their intention to use the app continuously.

People’s intrinsic needs fulfillment has also been associated with purchase intention (Huang et al., 2016), which can be elicited when individuals’ desire for competence and autonomy is gratified. For example, when consumers feel that they can use their abilities effectively (i.e., ‘competence’ need fulfillment) and have control over their own actions (i.e., ‘autonomy’ need fulfillment) while browsing an online retail store, the greater their satisfaction with their online shopping experience and purchase intention (Kim & Lee, 2020). Further, the more consumers experience enhanced connections (i.e., ‘relatedness’ need fulfillment) with an online retailer, the longer they stay at the retailer’s store and more likely they are to purchase from it. This led us to postulate:

##### H8

The more consumers feel that their needs for (a) competence, (b) autonomy and (c) relatedness are satisfied while using a VR fashion app, the greater their intention to make in-app purchases.

Lastly, we believe that consumers’ intention to use a VR app continuously will increase their intention to make in-app purchases. Many previous studies have documented the effect of continuous use intention on purchase intention. In a social media context, the more people used social media continuously, the more they showed the intention to purchase via social media (Cho et al., 2012). Similarly, the longer and more often people spent time on an online retail site, the more likely they were to purchase products on it (Rosen, 2001). This led us to propose:

##### H9

Consumers’ intention to use a VR fashion app continuously will have a significant and positive effect on their intention to make in-app purchases.

**Fig. 1:**
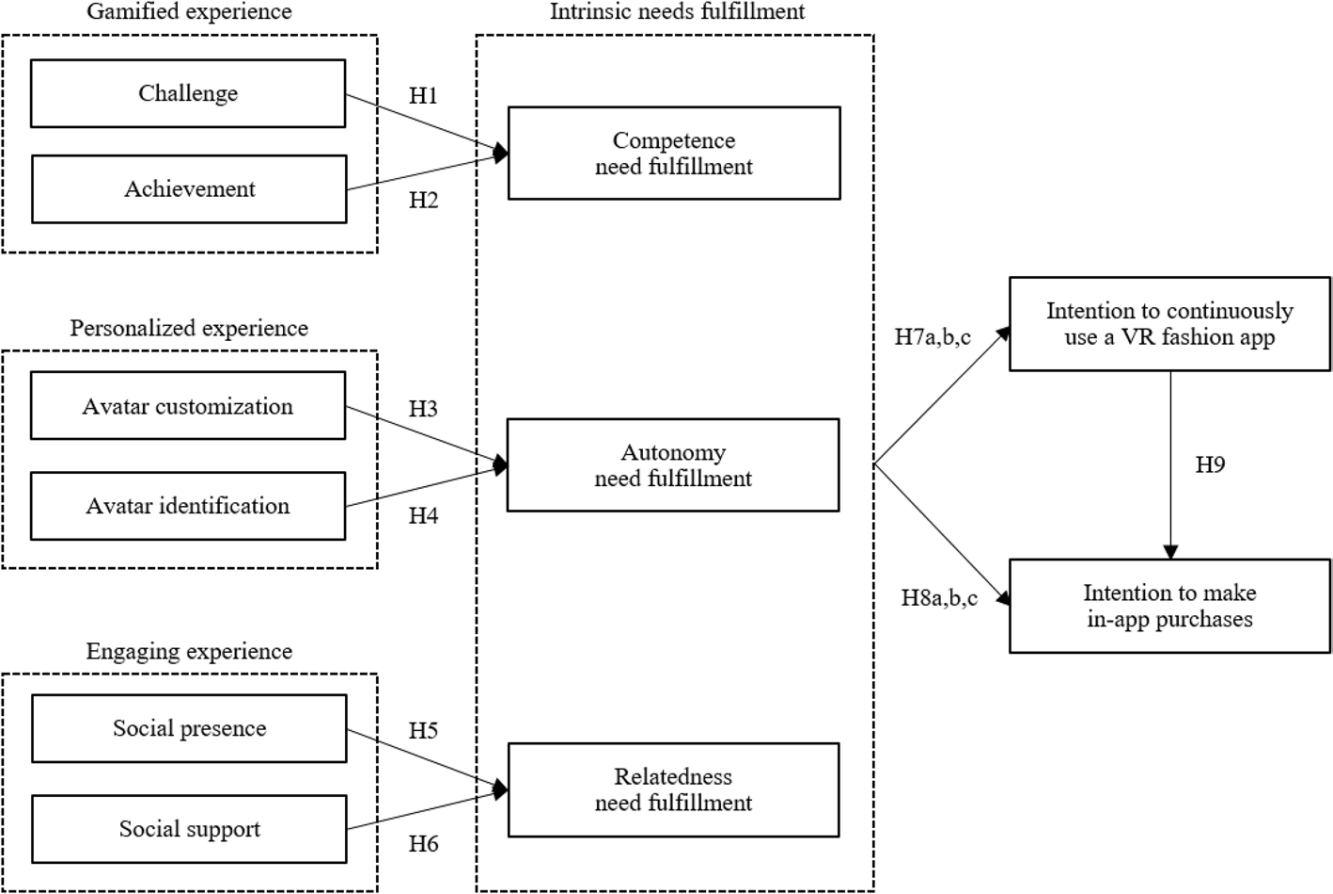
Research model

Figure 1 illustrates our research model and hypotheses.

## Methods

### Stimuli

**Fig. 2:**
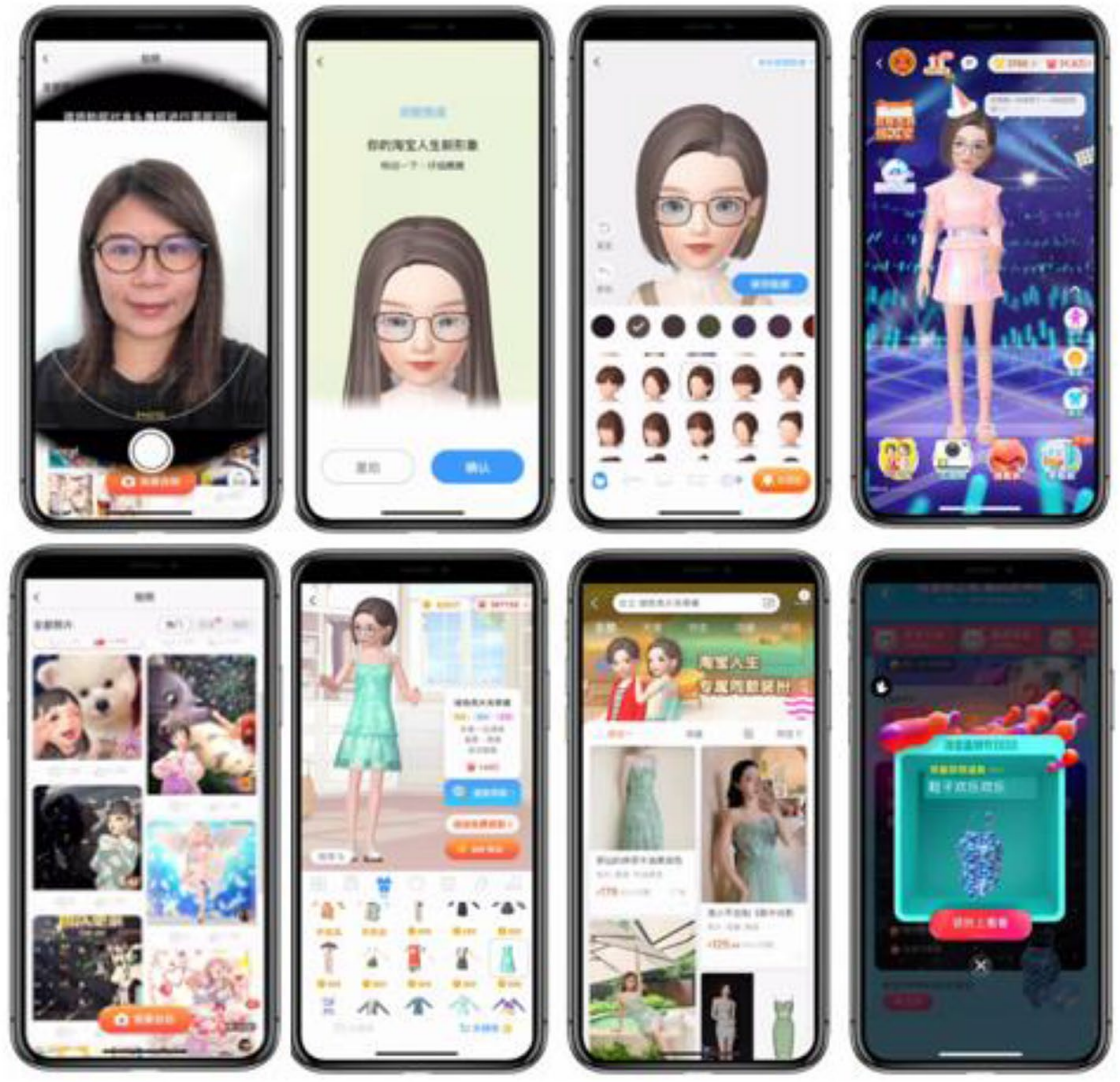
Stimuli: Taobao life, a 3D avatar-based and game-featured virtual world on the Taobao app

We measured consumers’ experiences with, and perceptions of, Alibaba’s Taobao Life. As shown in Fig. 2, Taobao Life is a 3D avatar-based virtual world integrated with the Taobao app. We chose Taobao Life as the main stimulus of a VR fashion app for three reasons. First, VR apps have surged in popularity recently because of the coronavirus outbreak, and Alibaba seized this opportunity by offering/promoting the VR Taobao Life on the Taobao app. Second, Taobao Life is integrated with China’s largest online retail platform (i.e., the Taobao app). Third, the distinct features embedded in Taobao Life were found to fit best to address this study’s purpose. In Taobao Life, consumers can navigate an immersive and game-like virtual world with their own customized 3D avatars. Every time customers spend money on Taobao, they receive points in Taobao Life that can be used to customize or style their personal avatars (Yu, 2020). For example, with the reward points, customers can shop for virtual items, such as digital clothing or VR accessories, some of which are based upon actual fashion items offered on Taobao (Alizila, 2020). To earn more points and buy more digital fashion items, customers can complete daily tasks, such as collecting the number of stars required by passing different levels of a tile-matching game on Taobao Life (Alizila, 2020). In Taobao Life, customers can also interact with other customers’ 3D avatars, take pictures with them, and post the pictures to a feed (Hallanan, 2020). Appendix shows additional features of Taobao life.

### Survey procedure

To test our research model and hypotheses, we developed an online survey questionnaire using Qualtrics. As Taobao Life is an app a Chinese retailer created, we chose Chinese consumers as our study sample. The online survey questionnaire consisted of three sections. In the first, we provided both visual and textual descriptions of Taobao Life to enhance survey participants’ understanding of our study’s context. To increase the data’s quality, we included a screening question (yes-or-no question) at the beginning of the questionnaire: “Have you played Taobao Life in the past three months?” Those who answered “no” to the question were screened out, while those who answered “yes” were asked to continue answering the rest of the questionnaire. In the second section, participants were asked to indicate their perceptions of, and behavioral intention toward, Taobao Life. In the last section, they were asked to provide their demographic information (gender, age, marital status, education, employment, and annual income). The survey was administrated through Dynata, a survey company that has a representative consumer panel in China. After a week of data collection, we collected 251 responses for data analysis, which fell under the rules-of-thumb: (1) A minimum sample size of 200 (Boomsma, 1982), and (2) 10 cases per variable (Nunnally & Bernstein, 1994). Furthermore, as shown in Table 1, our sample size n = 251 had 100% power (p < 0.05) to detect desired effect size of all structural paths in population, exceeding the suggested threshold, i.e., 80% (Cohen, 2013).

**Table 1:** Results of power analysis (*n* = 251)

Dependent variables			Independent variables	Number of predictors	Observed R^2^	Statistical power (%)
COM-N	←		CHA, ACI	2	.93	100
AUT-N	←		AVC, AVI	2	.85	100
REL-N	←		SOP, SOS	2	.90	100
CI	←		COM-N, AUT-N, REL-N	3	.70	100
PI	←		COM-N, AUT-N, REL-N, PI	4	.48	100

*CHA* Challenge, *ACI* Achievement, *AVC* Avatar customization, *AVI* Avatar identification, *SOP* Social presence, *SOS* Social support, *COM-N* Competence need fulfillment, *AUT-N* Autonomy need fulfillment, *REL-N* Relatedness need fulfillment, *CI* Intention to continuously use a VR fashion app, *PI* Intention to make in-app purchases

All power estimates were obtained from power analyses with sample size of *n* = 251 at alpha level = .05

According to our descriptive analysis, 31.9% of the respondents reported that they play Taobao Life every few days and 29.5% reported that they play it once a day, indicating the respondents’ high use of, and familiarity with, it. With respect to respondents’ demographic information, most were female (58.2%), aged between 26 and 40 (64.9%), who held a Bachelor’s degree (73.7%), and were married (72.9%). Further, the majority reported that they work full-time (89.2%), and 47% reported that they earn an annual income of more than 75,000 yuan. Details about our respondents’ demographics are presented in Table 2.

**Table 2:** Summary of the respondents’ sociodemographic profiles (*n* = 251)

Sociodemographic variables	Description	Frequency	Percentage
Gender	Male	105	41.8
	Female	146	58.2
Age	18 to 25	47	18.7
	26 to 40	163	64.9
	41 to 50	29	11.6
	51 to 70	12	4.8
Marital status	Single, never married	56	22.3
	Domestic partnership	9	3.6
	Married	183	72.9
	Separated, divorced, or widowed	3	1.2
Employment status	Work full-time	224	89.2
	Work part-time	13	5.2
	Multiple part-time jobs	2	0.8
	Do not work	12	4.8
Education level	High school or less	11	4.4
	Some college	36	14.3
	Bachelor’s degree	185	73.7
	Graduate degree	18	7.2
	Other	1	0.4
Annual disposable income	Less than 7500 yuan	13	5.2
	7500–15,000 yuan	28	11.2
	15,001–25,000 yuan	21	8.4
	25,001–40,000 yuan	19	7.6
	40,001–75,000 yuan	52	20.7
	75,001 yuan or more	118	47.0

### Measures

The measurement items in our questionnaire were adapted from previous studies and then translated into Chinese. The scale items for *challenge* were adapted from Fu et al. (2009); *achievement* from Li et al. (2015); *avatar customization* from Teng (2010); *avatar identification* from Moon et al. (2013) and Teng (2019); *social presence* from Mäntymäki and Salo (2010); *social support* from Ki et al. (2020); *competence need fulfillment* from Xi and Hamari (2019); *autonomy need fulfillment* from La Guardia et al. (2000); *relatedness need fulfillment* from Xi and Hamari (2019); *intention to continuously use a VR fashion app* from Merhi (2016), and *intention to make in-app purchases* from Kwahk and Kim (2017). All questions were answered on a 7-point Likert-type scale that ranged from *strongly disagree* (1) to *strongly agree* (7). Before the main study, we conducted a pilot test with 50 respondents and the results showed that they understood all items. Table 3 presents the instrument we used in our study.

**Table 3:** Measurement items and confirmatory factor analysis results (*n* = 251)

Measurement items in Chinese [translated into English]	Factor loading	AVE	CR
*Challenge*		.52	.81
淘宝人生的困难等级适合我。 [The level of difficulty in Taobao Life is suitable for me.]	.66		
我的技能在淘宝人生的游戏过程中逐渐地得到提高。 [My skills gradually improve through the course of the game in Taobao Life.]	.71		
技能的提高赋予我动力。 [I am motivated by the improvement of my skills.]	.77		
淘宝人生按照适当的速度提供新的挑战。 [Taobao Life provides new challenges at an appropriate pace.]	.76		
*Achievement*		.60	.86
我玩淘宝人生是为了达到更高的等级。 [I play Taobao Life to achieve a higher level.]	.73		
我玩淘宝人生是为了拥有比别人更大的权利。 [I play Taobao Life to have more power than others.]	.73		
我玩淘宝人生是为了获得装备和/或物品, 从而获得比其他游戏玩家更高的身份。 [I play Taobao Life to have the equipment and/or items, which give me higher status than other players in the game.]	.84		
我玩淘宝人生是为了向其他游戏玩家证明我是最棒的。 [I play Taobao Life to prove to other players in the game that I am the best.]	.81		
*Avatar customization*		.51	.75
淘宝人生允许用户为自己的形象定制装备、配饰和装饰。 [Taobao Life enables users to customize the equipment, accessories, and decorations of their avatars.]	.72		
淘宝人生允许用户定制自己形象的外观。 [Taobao Life enables users to customize the appearance of their avatars.]	.70		
淘宝人生允许用户为自己的形象创建定制的商品和装备。 [Taobao Life enables users to create customized goods and equipment of their avatars.]	.71		
*Avatar identification*		.71	.91
我对我在淘宝人生中的形象有一种强烈的拥有感。 [I have a strong feeling of ownership toward my avatar in Taobao Life.]	.85		
我觉得我在淘宝人生中的形象是我自己的延伸。 [I feel that my avatar in Taobao Life is an extension of myself.]	.86		
我在淘宝人生中的形象给了我一种自我表达的方式。 [My avatar in Taobao Life provides me a kind of self-expression.]	.84		
我在淘宝人生中的形象对我来说极为重要。 [My avatar in Taobao Life is extremely important to me.]	.81		
*Social presence*		.66	.85
淘宝人生中有一种人际交往的感觉。 [There is a sense of human contact in Taobao Life.]	.82		
淘宝人生中有一种社交感 (用户可以交友) 。 [There is a sense of sociability in Taobao Life (users are companionable).]	.80		
淘宝人生中有一种人性的温暖感。 [There is a sense of human warmth in Taobao Life.]	.82		
*Social support*		.69	.90
如果淘宝人生中的用户是真人, 我完全可以信任他们。 [If the users in Taobao Life were real people, I could trust them completely.]	.80		
如果淘宝人生中的用户是真人, 他们可以在需要的时候依靠我。 [If the users in Taobao Life were real people, they would be able to count on me in times of need.]	.86		
如果淘宝人生中的用户是真人, 我会给予他们情感上的支持。 [If the users in Taobao Life were real people, I would give them emotional support.]	.81		
如果淘宝人生中的用户都是真人, 我会在需要的时候依靠他们。 [If the users in Taobao Life were real people, I would be able to count on them in times of need.]	.84		
*Competence need fulfillment*		.67	.89
我玩淘宝人生 的时候, 我觉得自己是一个有能力的人。 [I feel like a competent person when I am playing Taobao Life.]	.84		
我对自己玩淘宝人生的表现很满意。 [I am satisfied with my performance when I am playing Taobao Life.]	.82		
我觉得自己是淘宝人生虚拟世界的专家。 [I feel like an expert in the virtual world of Taobao Life.]	.77		
我认为自己在玩淘宝人生的时候表现很棒。 [I think that I am pretty good when I am playing Taobao Life.]	.84		
*Autonomy need fulfillment*		.64	.90
当我浏览淘宝人生的时候, 是因为我想浏览。 [When I visit Taobao Life, it is because I want to visit it.]	.72		
当我玩淘宝人生的时候, 我可以随心所欲地做自己。 [When I am playing Taobao Life, I feel free to be who I am.]	.83		
当我玩淘宝人生的时候, 我觉得我可以做自己。 [I feel I can be myself when I am playing Taobao Life.]	.86		
当我玩淘宝人生的时候, 我拥有发言权, 可以发表自己的意见。 [When I am playing Taobao Life, I have a say in what happens and can voice my opinion.]	.84		
当我玩淘宝人生的时候, 我可以从外界的压力中解脱出来。 [I feel free from outside pressures when I am playing Taobao Life.]	.74		
*Relatedness need fulfillment*		.73	.91
当我玩淘宝人生的时候, 我感觉得到了其他用户的支持。 [When I am playing Taobao Life, I feel supported by other users.]	.87		
当我玩淘宝人生的时候, 我感觉自己得到了别人的理解。 [When I am playing Taobao Life, I feel that I am understood.]	.87		
当我玩淘宝人生的时候, 我感觉自己是一个对别人有价值的人。 [When I am playing Taobao Life, I feel that I am a valuable person to others.]	.84		
当我玩淘宝人生的时候, 我感觉别人留心我的所言所行。 [When am playing Taobao Life, I feel like other people care what I have to say and what I do.]	.83		
*Intention to continuously use a VR fashion app*		.83	.94
我打算以后一直玩淘宝人生。 [I intend to keep playing Taobao Life in the future.]	.88		
我打算继续玩淘宝人生。 [I intend to continue playing Taobao Life.]	.92		
我相信自己以后会玩淘宝人生。 [I believe I will play Taobao Life in the future.]	.94		
*Intention to make in-app purchases*		.49	.66
我想在淘宝商城上购物。 [I would like to purchase on Taobao app.]	.75		
我会考虑以后在淘宝商城上购物。 [I would consider purchasing on Taobao app in the future.]	.65		

*AVE* Average variance extracted, CR composite reliability

## Results

We conducted a structural equation model (SEM) analysis of the data the 251 respondents provided, to test our measurement and structural models, and hypotheses. The details of our SEM results are presented below.

### Measurement model evaluation results

The results of our measurement model evaluation using confirmatory factor analysis showed a satisfactory model fit: χ^2^_686_ = 1558.09, CFI = 0.90, TLI = 0.88, IFI = 0.90 and NFI = 0.83, and RMSEA = 0.07. To validate our measurements further, we tested our instrument’s convergent and discriminant validities. As shown in Table 3, all factor loadings were above 0.65, exceeding the 0.60 threshold value (Awang et al., 2015). Second, the average variance extracted (AVE) for each construct ranged from 0.51 to 0.83, greater than the 0.50 threshold (Hair et al., 2010). Third, all constructs’ composite reliabilities (CR) were between 0.66 to 0.94, greater than the 0.60 threshold value (Fornell & Larcker, 1981). Further, as shown in Table 4, the Heterotrait-monotrait (HTMT) ratio of correlations between all possible pairs of constructs was below the recommended threshold value of 0.90, which confirmed discriminant validity (Gold et al., 2001; Henseler et al., 2015).

**Table 4:** Heterotrait-monotrait (HTMT) ratio of correlation

Constructs	1	2	3	4	5	6	7	8	9	10
1. CHA	–									
2. ACI	.69	–								
3. AVC	.71	.56	–							
4. AVI	.80	.76	.65	–						
5. SOP	.86	.68	.66	.83	–					
6. SOS	.71	.64	.53	.74	.80	–				
7. COM-N	.84	.78	.70	.89	.88	.80	–			
8. AUT-N	.83	.68	.71	.89	.90	.76	.88	–		
9. REL-N	.81	.71	.59	.86	.89	.86	.88	.87	–	
10. CI	.84	.63	.62	.80	.79	.63	.80	.81	.76	–
11. PI	.57	.31	.62	.54	.48	.45	.51	.60	.49	.67

*CHA* Challenge, *ACI* Achievement, *AVC* Avatar customization, *AVI* Avatar identification, *SOP* Social presence, *SOS* Social support, *COM-N* Competence need fulfillment, *AUT-N* Autonomy need fulfillment, *REL-N* Relatedness need fulfillment, *CI* Intention to continuously use a VR fashion app, *PI* Intention to make in-app purchases

### Structural model evaluation and hypothesis test results

The results of our structural model evaluation also showed a satisfactory model fit: χ^2^_712_ = 1505.90, CFI = 0.91, TLI = 0.90, IFI = 0.91, NFI = 0.84, and RMSEA = 0.07. As shown in Table 5, our results show that all of the hypotheses but H7c, H8a and H8c were supported. Specifically, challenge (H1: β = 0.80, *p* < 0.01) and achievement (H2: β = 0.21, *p* < 0.01) affected *competence need fulfillment* significantly and positively; avatar customization (H3: β = 0.27, *p* < 0.01) and avatar identification (H4: β = 0.72, *p* < 0.01) affected *autonomy need fulfillment* significantly and positively, and social presence (H5: β = 0.67, *p* < 0.01) and social support (H6: β = 0.33, *p* < 0.01) affected *relatedness need fulfillment* significantly and positively. In turn, while competence need fulfillment (H7a: β = 0.48, *p* < 0.01) and autonomy need fulfillment (H7b: β = 0.40, *p* < 0.01) had a significant influence on continuous app use intention, relatedness needs fulfillment’s effects (H7c: β = 0.00, *p* > 0.05) on continuous use intention were insignificant. With respect to the determinants of in-app purchase intention, competence need fulfillment (H8a: β = − 0.29, *p* > 0.05) and relatedness need fulfillment (H8c: β = − 0.02, *p* > 0.05) had no significant effect on in-app purchase intention. Only autonomy need fulfillment (H8b: β = 0.41, *p* < 0.05) affected in-app purchase intention significantly and positively. Lastly, the intention to use a fashion VR app continuously had a significant and positive effect on the in-app purchase intention (H9: β = 0.59, *p* < 0.01).

**Table 5:** Structural model evaluation and hypothesis test results (*n* = 251)

Hypothesis	Structural path	*β*	*t*-value	Result
H1	CHA → COM-N	0.80***	9.04	Supported
H2	ACI → COM-N	0.21***	3.25	Supported
H3	AVC → AUT-N	0.27***	4.15	Supported
H4	AVI → AUT-N	0.72***	9.51	Supported
H5	SOP → REL-N	0.67***	8.64	Supported
H6	SOS → REL-N	0.33***	4.51	Supported
H7a	COM-N → CI	0.48***	4.05	Supported
H7b	AUT-N → CI	0.40***	4.18	Supported
H7c	REL-N → CI	0.00	0.03	Not supported
H8a	COM-N → PI	− 0.29	− 1.45	Not supported
H8b	AUT-N → PI	0.41**	2.55	Supported
H8c	REL-N → PI	− 0.02	− 0.14	Not supported
H9	CI → PI	0.59***	4.14	Supported

Fit statistics	
χ^2^_df_	χ^2^_712_ = 1505.90
CFI	.91
TLI	.90
IFI	.91
RMSEA	.07

*CHA* Challenge, *ACI* Achievement, *AVC* Avatar customization, *AVI* Avatar identification, *SOP* Social presence, *SOS* Social support, *COM-N* Competence need fulfillment, *AUT-N* Autonomy need fulfillment, *REL-N* Relatedness need fulfillment, *CI* Intention to continuously use a VR fashion app, *PI* Intention to make in-app purchases

^**^p < .05 ***p < .01

## Discussion

While retailers are developing VR fashion apps that integrate gamification, personalization, and engagement elements increasingly as a way to cope with the COVID-19 pandemic and promote online shopping, less is understood about whether this novel type of VR fashion app increases fashion retailers’ online sales (van Heerde et al., 2019). This study sought to address this gap in the literature by adopting SDT within the context of Taobao Life. Our research findings provide several theoretical implications to the fashion mobile app literature and practical implications that can be applied in fashion marketers’ m-commerce strategies.

Our research provides new insights to the literature by shedding light on consumers’ ‘intrinsic needs fulfillment’ (i.e., competence, autonomy, and relatedness needs fulfillment) as the essential antecedents that motivate their VR fashion app use and in-app purchase behaviour. While the theory of reasoned action has influenced previous research largely and thus asserted that ‘consumer attitude’ is an important factor that predicts consumer behaviour (Choi & Lee, 2019; Kim & Karpova, 2010; Kim et al., 2016; Rauschnabel et al., 2019), less is understood about whether, and if so, the way consumers’ ‘intrinsic needs fulfillment’ influences their VR app use and in-app purchase behaviour. Understanding this is important, as recent studies have documented that SDT’s autonomous motivation predicts consumer behaviour better than its more established predictors (e.g., ‘attitude’) (Gilal et al., 2018, 2019; Xi & Hamari, 2019). While accounting for the critical role a positive attitude plays in eliciting desirable consumer behavior, such as consumer adoption and satisfaction (Hur et al., 2017; Trivedi & Trivedi, 2018), our findings show that the extent to which consumers feel their inherent needs for competence, autonomy, and relatedness are fulfilled is critical in motivating their behavioral intention to use VR fashion apps continuously and make in-app purchases. Thus, to maximize the significant benefits VR fashion apps can offer, it is important for retailers and marketers to develop in-app features that can satisfy consumers’ strong intrinsic needs for competence, autonomy, and relatedness.

Our research findings provide further insights into this aspect by identifying the specific factors that affect consumers’ competence, autonomy, and relatedness needs fulfillment, which in turn, influence their app use and in-app purchases. Two factors were found to be important to fulfill VR fashion app consumers’ *competence* need. Our findings show that the more consumers experience a sense of challenge (i.e., the perception of facing new challenges in the VR game world) and achievement (i.e., the perception of success by reaching a higher level) while using a VR fashion app, the more they feel that their need for competence is gratified. This indicates that fashion retailers and market practitioners could benefit by enhancing VR fashion apps’ game features that can enhance consumers’ sense of challenge and achievement, which in turn, satisfy their intrinsic need for competence. As for consumers’ need for *autonomy*, our findings show that fulfilling the need for autonomy is enhanced when consumers experience VR fashion apps’ customization features. The more consumers were given the freedom to personalize their digital avatars and thus, were able to perceive that the avatars are similar to them, the more they felt that their need for autonomy was gratified. On the other hand, consumers experienced fulfillment of their need for *relatedness* when they felt a sense of social presence and social support while using VR apps. According to our findings, the more consumers felt a sense of human contact while playing VR fashion apps, the more it satisfied their need for relatedness. Further, the more they felt that they could exchange emotional support with other users/players in a VR fashion app, the more their need for relatedness was fulfilled as well.

Our research findings show that it is this fulfillment of inherent needs (i.e., competence and autonomy) that motivates consumers’ behaviors. With respect to the factors that affect consumers’ continuous VR fashion app use intention, it was found important to fulfill two needs. The more consumers felt that their needs for competence and autonomy were satisfied, the more they were motivated to use VR fashion apps continuously. However, consumers’ need for relatedness was not found to have a significant effect on their intention to use apps continuously. This implies that the VR fashion app usage behavior is more influenced by the gamified and personalized app features, which in turn, lead to consumers’ competence and autonomy needs fulfillment, respectively. These findings infer that when it comes to VR fashion app usage, the fulfill of consumers’ needs for competence and autonomy has positive effect on their app usage behavior; but not the fulfill of their need for relatedness. This may be because relatedness need can be fulfilled through other human–computer interactions (e.g., their use of social media or online games), not necessarily through the use of VR fashion apps.

With respect to in-app purchase intention, only fulfilling the need for autonomy was found to be a significant antecedent. This supports previous research findings that indicate autonomy as a critical aspect of consumer choice decisions (André et al., 2018; Wertenbroch et al., 2020). While fulfilling competence and relatedness needs did not affect consumers’ in-app purchase intention, our results show that the more consumers fulfill their need for autonomy by customizing their 3D avatars’ visual features, such as the face, hairstyle, and clothing, to resemble their own, the greater their propensity to make in-app purchases. Furthermore, the more consumers use VR fashion apps continuously, the greater their propensity to make in-app purchases. These findings imply that to maximize their mobile sales, fashion retailers and m-commerce marketers may wish to focus on fulfilling consumers’ need for autonomy by enhancing their VR fashion apps’ avatar customization and identification features (e.g., features that allow customers to virtually dress their avatars and to project some aspects of their real-life identities onto those avatars), and in the long-term, by motivating customers to use their VR fashion apps continuously.

## Conclusions

This research was designed to address the question: can VR fashion apps increase online sales? Our research findings show clearly that fashion retailers’ VR apps indeed can have a positive marketing effect by fulfilling consumers’ inherent needs for competence and autonomy by employing their apps’ gamified (i.e., challenge and achievement) and personalized features (i.e., avatar customization and identification). In this way, our study provides important, new insights to the literature by highlighting the two needs that must be fulfilled to motivate consumers’ intention to use VR fashion apps continuously and/or make in-app purchases.

## Data Availability

The data that support the findings of this study are available on request from the corresponding author, Dr. Chung-Wha (Chloe) Ki. The data are not publicly available due to their containing information that could compromise the privacy of research participants.

## Author’s information

Oiyan Lau, a master student, and Dr. Chung-Wha (Chloe) Ki, an assistant professor, at Hong Kong Polytechnic University.
